# MCP arthrodesis using an intramedullary interlocking device

**DOI:** 10.1007/s11552-013-9579-5

**Published:** 2013-12-05

**Authors:** Jacqueline C. Vanderzanden, Brian D. Adams, Justin J. Guan

**Affiliations:** 1 Department of Orthopedic Surgery and Rehabilitation, University of Iowa, Iowa City, IA 52242 USA; 2 New England Orthopedics, Baystate Medical Center, 300 Birnie Ave, Suite 201, Springfield, MA 01107 USA; 3 Department of Orthopedics and Rehabilitation, University of Iowa Hospitals and Clinics, Iowa City, IA 52242 USA

**Keywords:** Metacarpophalangeal joint, MCP, MCPJ, Fusion, Joint fusion, Arthrodesis, Arthritis, Osteoarthritis, Rheumatoid arthritis

## Abstract

**Background:**

A variety of metacarpophalangeal joint (MCPJ) arthrodesis techniques have been described for the treatment of symptomatic arthritis and instability of the thumb MCPJ including K wire fixation, tension-band arthrodesis, plate fixation, intramedullary screw, and other intramedullary devices. This study presents a retrospective review of one surgeon's initial series of patients undergoing thumb MCP arthrodesis using an intramedullary compression device with a fixed angle of 25°.

**Methods:**

A retrospective chart and radiographic review of patients treated for thumb MCP arthrodesis using the intramedullary device was performed. Final radiographs were evaluated for arthrodesis angle, bony fusion, and implant fixation. Any complication found during surgery or the follow-up period was noted.

**Results:**

In this study, 17 patients were reviewed. Indications for surgery were osteoarthritis (five patients), rheumatoid arthritis (three patients), MCP instability alone (seven patients), and post-traumatic conditions (two patients). Of these, 12 patients had a simultaneous trapeziometacarpal (TMC) soft tissue arthroplasty. Mean follow-up was 4.9 months. All 17 patients had clinical and radiographic evidence of fusion at an average of 7.9 weeks, with an average fusion angle of 24.4°. There were no hardware complications, no infections, no revisions, and no indications for hardware removal.

**Discussion:**

Our study results indicate the technique promotes rapid union at a precise angle, provides strong fixation that does not require prolonged immobilization, does not cause hardware irritation, and can be used in conjunction with other procedures including TMC arthroplasty when MCP arthrodesis is indicated for joint instability.

## Introduction

Symptomatic osteoarthritis of the thumb metacarpophalangeal joint (MCPJ) is far less common than that of the trapeziometacarpal joint (TMCJ) [7]; however, MCPJ instability associated with TMC arthritis and rheumatoid arthritis affecting the MCPJ are common indications for MCP arthrodesis [1]. In addition, chronic post-traumatic instability with or without arthritis and failed collateral ligament repairs are also indications for MCP arthrodesis.

**Fig. 1:**
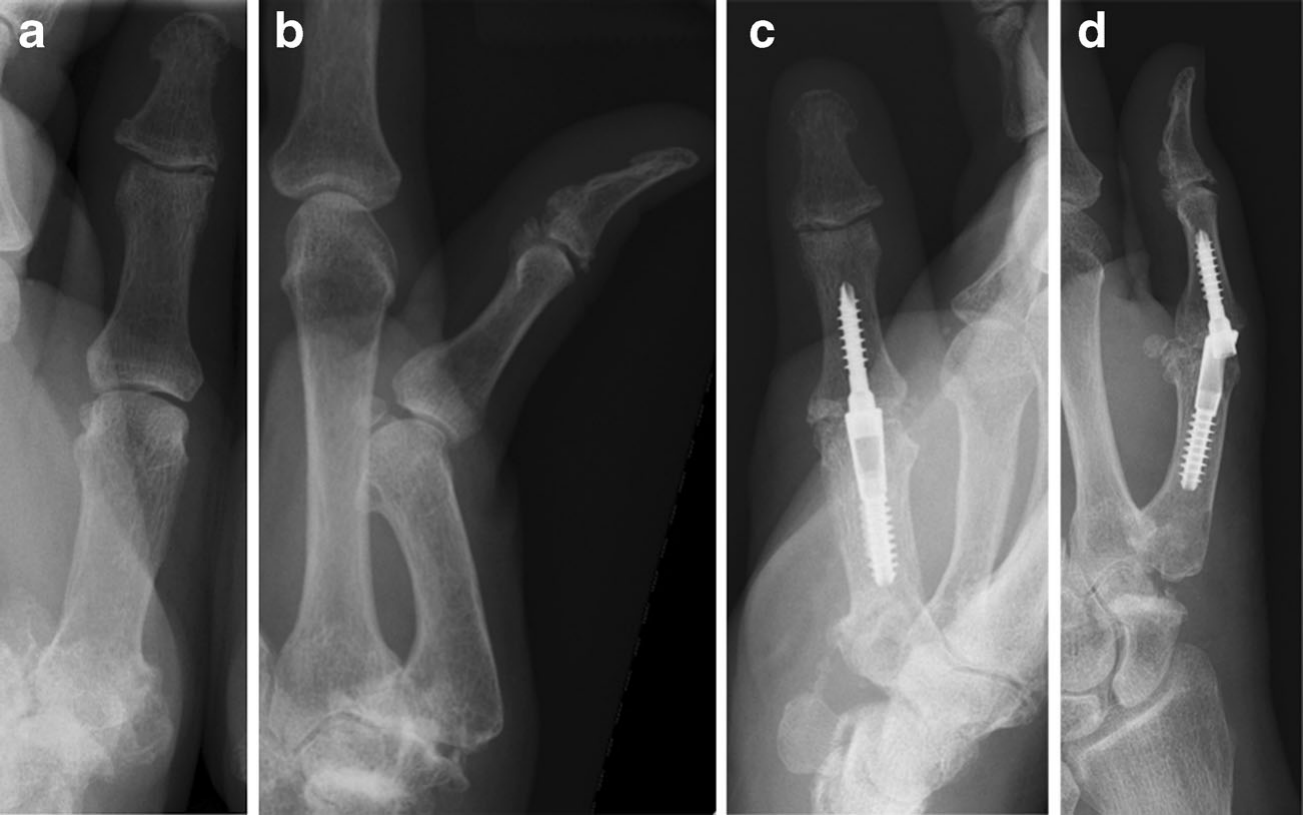
**a** Preoperative AP radiograph, left thumb of 60 yo female with trapeziometacarpal osteoarthritis and metacarpophalangeal joint instability (Pt. 6). **b** Preoperative lateral radiograph, left thumb of Pt. 6. **c** Postoperative (4 weeks) AP radiograph, left thumb of Pt. 6 showing intramedullary compression device and fusion of metacarpophalangeal joint. Postoperative (4 weeks) lateral radiograph, left thumb of Pt. 6 showing intramedullary compression device and fusion of metacarpophalangeal joint

**Fig. 2:**
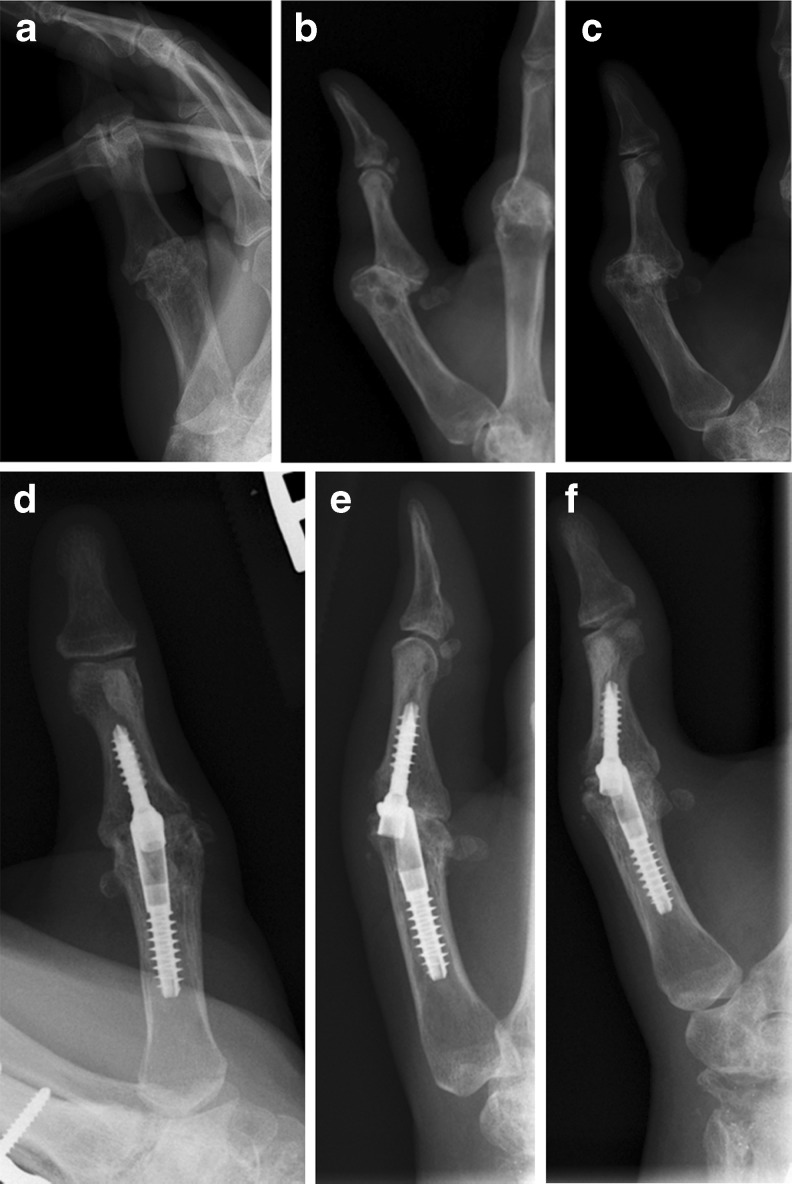
**a** Preoperative AP radiograph, right thumb of 40 yo female with rheumatoid arthritis and metacarpophalangeal joint instability and arthritis (Pt. 9). **b** Preoperative lateral radiograph, right thumb of Pt. 9. **c** Preoperative oblique radiograph, right thumb of Pt. 9. **d** Postoperative (5 weeks) AP radiograph, right thumb of Pt. 9 showing intramedullary compression device and fusion of metacarpophalangeal joint. **e** Postoperative (5 weeks) lateral radiograph, right thumb of Pt. 9 showing intramedullary compression device and fusion of metacarpophalangeal joint. **f** Postoperative (5 weeks) oblique radiograph, right thumb of Pt. 9 showing intramedullary compression device and fusion of metacarpophalangeal joint

A variety of surgical techniques and fixation devices have been described for MCP arthrodesis [1, 4–9]. K wires with or without a tension band is a common technique [1, 4, 7–9]. Several intramedullary screw fixation methods have also been used in an attempt to provide stronger fixation and less hardware irritation than K wires [1, 4–6]. The characteristics of an optimal technique for MCP arthrodesis would include sufficient fixation to allow early thumb mobilization, promotes rapid fusion, and applicable for all arthrodesis indications. The recommended angle of fusion is typically between 10° and 32°, which is based on studies that assessed MCP joint motion used in activities of daily living [2, 7, 11].

The goal of this study is to assess the clinical and radiographic outcomes of a single surgeon's initial experience using a fixed angle intramedullary compression device (Extremity Medical, Parsippany, NJ) (Figs. 1 and 2).

## Materials and Methods

The institutional review board of the authors' institution approved this retrospective chart and radiographic review of an initial series of patients treated by thumb metacarpophalangeal arthrodesis using the intramedullary device. The patients were treated over a 3-year period. Preoperative lateral, posteroanterior (PA), and oblique radiographs of the thumb were used to assess for MCP arthritis and joint alignment. Final radiographs were evaluated for arthrodesis angle based on the lateral view, bony fusion, and implant fixation based on the lateral, PA, and oblique views, with fusion judged as bone bridging across the fusion site [6].

### Surgical Technique

A dorsal longitudinal midline incision is made over the thumb MCPJ. The extensor mechanism is incised longitudinally in its midline. The joint capsule and periosteum over the metacarpal and proximal phalanx are also incised longitudinally and reflected radially and ulnarly. The collateral ligaments are released from the metacarpal head to provide full joint flexion and complete exposure of the articular surfaces.

Preoperative X-ray templating is used to determine appropriate implant size. A guide wire is inserted into the center of the metacarpal medullary canal and its position confirmed with fluoroscopy. A cannulated drill followed by a cannulated reamer is used to prepare the metacarpal canal. The selected metacarpal implant is inserted to the appropriate depth and rotation. A dual-purpose reamer and rasp is inserted through the implant to create a hole in the dorsal metacarpal neck and produce a flat metacarpal head with exposed cancellous bone at a preset 25° angle. The implant provides fusion only at 25° and does not allow for angle adjustments.

A guidewire is inserted down the center of the proximal phalanx medullary canal to a depth just beyond its isthmus, and the position is confirmed using fluoroscopy. A dual-purpose drill and rasp is used to prepare the canal and create a flattened head surface with exposed cancellous bone. A cannulated depth gauge is used to determine lag screw length. The lag screw is inserted through the dorsal window in the metacarpal implant and into the proximal phalanx canal. While the phalanx is held against the metacarpal head in proper rotation, the lag screw is inserted into the phalanx. Increasing torque is felt as the screw advances, first creating bony compression at the fusion site followed by engagement of the locking mechanism between the two components. Fluoroscopy is used to confirm screw position and bony compression. The capsule and extensor mechanism are closed sequentially using running absorbable sutures, followed by skin closure. The total procedural tourniquet time is approximately 30 min, depending on thumb deformity. Postoperative immobilizaon and care varied, which depended upon other procedures performed; however, all patients were initially placed into a thumb spica plaster splint, with transition to a short-arm thumb spica cast or removable splint at the first postoperative appointment at 2 weeks. By the 1-month postoperative appointment, all patients had been transitioned into a removable thumb spica splint and allowed to begin range of motion exercises of the thumb TMC and interphalangeal joint. Full activities were permitted at 2 months if other treated conditions allowed.

## Results

Surgical indications included TMC and MCP osteoarthritis with MCP instability (four patients), TMC osteoarthritis with MCP instability (five patients), TMC and MCP osteoarthritis (one patient), MCP instability alone (two patients), rheumatoid arthritis (three patients), chronic post-traumatic MCP arthritis and instability (one patient), and chronic post-traumatic MCP instability (one patient). Only one patient had isolated thumb MCP arthrodesis. The most common concomitant procedure was TMC arthroplasty (12 patients), which included trapeziectomy and soft tissue reconstruction. One patient underwent revision TMC arthroplasty as a concurrent procedure. One patient with rheumatoid arthritis underwent silicone implant arthroplasty of the 2^nd^–5^th^ MCP joints, one patient had tendon transfers for radial nerve palsy, one rheumatoid patient underwent removal of a wrist implant and wrist arthrodesis, and one patient underwent total wrist arthrodesis with distal ulna resection. Other concurrent soft tissue procedures included carpal tunnel release (2 patients), trigger thumb release (2 patients), and De Quervain's release (1 patient). 15 patients were female and two patients were male, with an average age of 58.7 years (range 40–75 years; Table 1).

**Table 1:** Patient gender, age, affected side, indications for MCP arthrodesis, concurrent procedure(s) performed, fusion angle, and time to radiographic fusion

Patient	Sex	Age	Side	Indication for MCP arthrodesis	Additional procedure(s)	Fusion angle	Time to radiographic fusion
1	F	57	Left	TMC OA with MCPJ instability and OA	TMC arthroplasty	24°	6 weeks
2	F	59	Left	TMC OA with MCPJ instability and OA	TMC arthroplasty, Carpal tunnel release	24°	12 weeks
3	F	68	Right	TMC OA with MCPJ instability and OA	TMC arthroplasty, DeQuervain's release	25°	11 weeks
4	F	57	Right	Chronic post-traumatic MCPJ instability and MCPJ OA	None	25°	11 weeks
5	F	55	Right	Rheumatoid arthritis with MCPJ instability and arthritis	TMC arthroplasty	25°	20 weeks
6	F	60	Left	TMC OA with MCPJ instability	TMC arthroplasty	23°	4 weeks
7	F	45	Left	TMC OA with MCPJ instability	Revision TMC arthroplasty, Trigger thumb release	25°	8 weeks
8	F	69	Right	Rheumatoid arthritis with MCPJ instability and arthritis	MCP silicone arthroplasties for digits 2–5	23°	8 weeks
9	F	40	Right	Rheumatoid arthritis with MCPJ instability and arthritis	Wrist arthrodesis	25°	5 weeks
10	F	75	Left	MCPJ instability	TMC arthroplasty, Tendon transfers for radial nerve palsy	23°	4 weeks
11	F	62	Right	TMC OA with MCPJ instability and OA	TMC arthroplasty	28°	4 weeks
12	M	53	Left	TMC and MCPJ OA	TMC arthroplasty, Trigger thumb release	24°	8 weeks
13	M	54	Right	Chronic post-traumatic MCPJ instability	TMC arthroplasty revision	25°	5 weeks
14	F	50	Right	TMC OA with MCPJ instability	TMC arthroplasty, Carpal tunnel release	25°	5 weeks
15	F	61	Right	TMC OA with MCPJ instability	TMC arthroplasty	24°	11 weeks
16	F	70	Right	TMC OA with MCPJ instability	TMC arthroplasty	24°	6 weeks
17	F	63	Right	MCPJ instability	Total wrist arthrodesis, distal ulna resection	23°	5 weeks

*MCP* metacarpophalangeal, *MCPJ* MCP joint, *OA* osteoarthritis, *TMC* trapeziometacarpal, *M* male, *F* female

Follow-up period ranged from 5 weeks to 23 months, with a mean of 4.9 months. All 17 patients achieved stable, pain-free MCP fusion as determined by clinical evaluation at final follow-up. The average fusion angle was 24.4° (range 23–25). The average time to fusion was 7.9 weeks based on radiographs obtained between 6 and 20 weeks postoperatively. While 16 patients had radiographs between 4 and 12 weeks, one patient had radiographs at 4 and 20 weeks, with the 20-week radiograph showing fusion. There were no complications related to the MCP arthrodesis including no cases of implant migration, implant breakage, peri-implant bone resorption, or implant removal.

## Discussion

Although isolated symptomatic MCP arthritis of the thumb is not a common condition that is indicated for surgery, MCP arthrodesis is a conventional procedure performed in combination with other procedures, particularly, TMC arthroplasty when excessive MCP hyperextension is present, and it is also performed for rheumatoid disease and some post-traumatic conditions.

A wide range of techniques has been used for MCP arthrodesis. K wire fixation is the traditional technique; however, problems with failure of fusion, pin tract infections, and hardware irritation that require implant removal are common [1, 7, 8]. Stanley et al. presented a series of 42 thumb MCPJ fusions using K wire fixation with an average follow-up of 22.5 months. Fusion was attained in 35 (83 %), with four cases having residual pain, one of which was mobile, and an additional four painless nonunions. Extensor pollicis longus tendon rupture occurred in two patients, and one patient had persistent operative site swelling. A comparison study between K wire and tension-band arthrodesis showed that K wire fixation led to infections in 18 % of cases, half of which contributed to malunions [3]. The rate of revision arthrodesis for the K wire fixation group approached 15 %. The tension-band arthrodesis group had a 95 % successful fusion rate, with 2 % having infections. In a separate study done by Uhl et al. [9] the fusion rate for tension-band arthrodesis approached 99 %, but 17 of 72 cases developed painful hardware after initial surgery, most of which required hardware removal. The average time to fusion was 12 weeks (range 4–64 weeks).

Plate fixation has been used to provide stronger fixation than K wires and to reduce the need for cast immobilization. A high fusion rate of 96 % was reported in one series, but there was a 15 % rate of plate removal due to hardware irritation and the infection rate was 6 % [10]. In their series, the angle of fusion of the MCPJ ranged from 10° to 15°, and the radiographic time to fusion was approximately 6 weeks.

Another option for arthrodesis is cannulated screw and threaded washer, which was evaluated by Schmidt et al. [6] in a series of 26 patients with a follow-up of 32 months. Stable, pain-free MCP fusion was achieved in 25 patients with no hardware failures, no indications for hardware removal, and no infections. While 11 patients had minor collapse surrounding the fixation screw, nine patients had bone resorption at the fusion site. The average fusion angle was 18° (range 0–38°), and average time to fusion was 10 weeks.

Savvidou and Kutz [5] reported a 16-month follow-up of seven patients treated with an intramedullary X-shaped implant for finger and thumb joint arthrodesis. The implant is available in different sizes and angles and provides a finite amount of compression as the barbs on the prongs expand after insertion. In this series, only one patient had a thumb MCP arthrodesis. A stable, painless fusion was achieved in six patients, with one developing a painless nonunion and one showing mild dorsal cortex erosion. The average fusion time was 11 weeks (range 8–15 weeks).

Our retrospective case series of 17 thumb MCP arthrodeses using an intramedullary fixed-angle compression device was intended to assess if the device attained its design objectives to provide versatility and strong fixation and avoid hardware irritation and prolonged immobilization, while it provides reliable compression to promote rapid fusion at a precise angle. Unlike the wide range of fusion angles found using K wire, tension-band, and screws, all 17 of our patients achieved a pain-free MCP arthrodesis at a consistent fusion angle. There were no cases of infections, no indications for hardware removal or revision MCP surgery, and no peri-implant resorption or loosening. The average time to MCP fusion in our patients was 7.9 weeks based on X-rays taken between 6 and 20 weeks, although the time to fusion may have been shorter if X-rays were routinely obtained earlier than 12 weeks. Early in the series, we tended to immobilize the thumb longer following surgery, but patients treated later in the series and subsequent to this study were rarely immobilized longer than 2 weeks before being transitioned to a removable thumb spica splint and initiation of thumb motion exercises. The change in postoperative care was based on our confidence in the fixation strength found at surgery and the findings at the initial 2-week postoperative follow-up, which showed rapid wound healing, minimal pain, and no evidence of hardware loosening. Various angles of MCP arthrodesis have been recommended in previous publications, with 25° being within the recommended range [1, 2, 7, 11].

Although the results from our clinical experience are excellent, the follow-up averaged only 4.9 months. However, due to the rigidity of the implant, long-term complications are not likely to occur with the exception of the potential for peri-implant bony fracture. Since none of our patients had severe joint destruction, it is unknown whether the device can be used in thumbs with severe joint deformity or substantial bone loss. Even though some of our patients achieved good fixation despite having some degree of osteopenia based on standard X-rays, we do not know the lower limit of bone quality that can be accepted when using this implant.

We conclude this device provides a reliable method for MCP arthrodesis for a variety of indications. Device insertion is not technically demanding even when performed in conjunction with other procedures on the thumb. The intramedullary location of the device also avoids soft tissue irritation and the subsequent need for implant removal. Bony fusion is rapid and consistent, and rehabilitation is simplified due to the device's strong fixation.
